# Understanding and Deterring Online Child Grooming: A Qualitative Study

**DOI:** 10.5964/sotrap.13147

**Published:** 2024-02-15

**Authors:** Sarah Wefers, Therese Dieseth, Emily George, Ida Øverland, Jayeta Jolapara, Ciara McAree, Donald Findlater

**Affiliations:** 1 The Lucy Faithfull Foundation, Epsom, United Kingdom; 2 Policing Institute for the Eastern Region (PIER), Anglia Ruskin University, Cambridge, United Kingdom; Department of Psychiatry and Psychotherapy, University Medical Center Mainz, Mainz, Germany

**Keywords:** online grooming, online offending, child sexual exploitation, prevention, deterrence

## Abstract

The prevalence of online child sexual grooming offenses has been on the rise, posing significant risks to children. Child sexual grooming involves sexual communication with minors. This study aims to understand motivations and pathways of individuals who have engaged in online grooming behaviour, as well as propose effective prevention and intervention strategies. A sample of 14 male participants who had engaged in online child grooming were interviewed. Five themes were identified through thematic analysis: Social aspects, Mental health and psychological aspects, Justification for offending, Secrecy and Technology. Within all five themes risk factors and protective factors relating to online grooming behaviour were identified. Three pathways into online grooming behaviour were hypothesised: social connection, addictive behaviour, and fantasy/roleplay. Additionally, the study highlights the complex relationship between online grooming and other child sexual abuse offences, including the sharing and distribution of indecent images of children. The study underscores the need for greater education and awareness about the risks and harms associated with online grooming for those at risk of engaging in this behaviour as well as wider support and situational prevention approaches, including monitoring and use of warning messages on relevant online platforms.

## Abstract (plain language summary)

**Background:**

Online sexual offences against children, including so-called “grooming” offences, have been increasing over recent years, particularly during the Covid-19 pandemic. These offences have severe negative impact on the children involved, the adults perpetrating the offences, and wider society.

**Why was this study done?:**

This study tried to deepen our understanding why and how grooming offending happens and what can be done to help people stop this behaviour and seek help. This was done to ultimately help reduce the number of grooming offences and keep more people safe online.

**What did the researchers do and find?:**

We interviewed 14 men who had been detected for their online sexual communications with minors and asked them about their pathways into offending and what they felt could be helpful in preventing grooming behaviour. We analysed the interviews and identified five themes that all related to both risk factors and protective factors for grooming behaviour. We named these themes Social Aspects, Mental Health/Psychological Aspects, Justification for Offending, Secrecy, and Technology. Participants spoke about how they used online chats to seek connections, how they felt their online behaviour got out of control, how they did not perceive online chats as ‘real’ conversations and that anonymity on the internet facilitated online grooming behaviour.

**What do these findings mean?:**

The findings show that there are different reasons why some men engage in online grooming behaviour. We propose that there may be three pathways into grooming offending, namely seeking intimacy and social connections; addictive behaviours; and fantasy. There are different things that can be done to prevent this behaviour, including more education about the risks of online behaviour, the use of warning messages online, and helping people lead a positive and healthy lifestyle.

## Highlights

- Social aspects, mental health issues, internal justifications, secrecy and the use of technology are central to understanding online grooming.
- Prevention of online grooming should include situational prevention on online platforms and education of the risks and harms of online behaviours.
- People at risk of online grooming behaviour should be supported in building positive offline relationships, addressing mental health needs and challenging offence-supportive cognitions.

Online child grooming is defined as the ‘act of developing a relationship with a child to enable their abuse and exploitation both online and offline’ (CEOP, n. d.). Online child grooming offences have increased steeply over the last years. According to the National Society for the Prevention of Cruelty to Children (NSPCC, 2021), the amount of detected online child grooming offences in the United Kingdom (UK) has increased by 70% from 2017/2018 to 2020/2021. Specifically, during Covid-19 lockdowns, reports about online grooming increased (NSPCC, 2020). However, offences that come to the attention of law enforcement are believed to only be the ‘tip of the iceberg’ (Beier et al., 2009). Therefore, the prevalence of offences like online child grooming is assumed to be much higher than those recorded by law enforcement agencies. A recent epidemiological study conducted in Spain found that 23% of teenagers aged 12-15 experienced sexual solicitation by an adult online (Ortega-Barón et al., 2022). This indicates that a large proportion of children are currently at risk of being groomed online. Being groomed online can have serious and severe consequences for children, including reduced health-related quality of life (Ortega-Barón et al., 2022), depression, self-harm and a negative impact on social relationships (Whittle et al., 2013).

Due to the serious and harmful nature of online child grooming offences and its impact on children, research is needed to understand this type of offending. Previous research has examined the online interactions between offenders and victims, identifying common stages in the conversations (O’Connell, 2003; Williams et al., 2013). These stages include rapport or friendship building, introducing sexual topics, and assessing and avoiding the risk of detection. Although this offers some insight into the conversations that may be taking place, most research is based on transcripts of conversations with decoy police officers (Joleby et al., 2021) and do not take into account the offender’s account, characteristics and circumstances at the time of offending.

Individuals who engage in online grooming appear to be a heterogenous group with diverse motivations (Taylor, 2017). Individuals who exploit children online have been identified to use their online behaviours to meet needs for intimacy or as a way to regulate negative emotions (Kloess et al., 2014; Middleton et al., 2006). Briggs et al. (2011) distinguished online-initiated child sexual offences as fantasy- versus contact-driven, where fantasy-driven individuals achieve gratification through online sexual behaviours and contact-driven individuals do so through contact sexual offending. Paquette and colleagues (2023) compared individuals who engaged in online grooming to users of child sexual exploitation material on various background and psychological variables. Their research indicated comparatively more considerable criminal histories and offence-supportive cognitions in those who had engaged in online grooming. Despite the growing prevalence of online grooming, research on individuals who have engaged in such behaviour, their pathways into offending and psychological characteristics is, however, still scarce. In addition, the ever-changing online landscape that constantly provides new avenues for offenders to get into contact with children and groom and exploit children in new ways (Westlake, 2019), poses challenges for research as findings may quickly become dated.

The current study therefore investigates recent online child grooming behaviour. The aim of this study is to understand the behaviour employed by individuals who have engaged in online grooming to inform deterrence strategies to prevent first-time and repeated online child grooming offences. Since 2015, the UK’s Lucy Faithfull Foundation (LFF) has run deterrence campaigns to raise awareness for online child sexual exploitation and abuse and encourage people who have engaged in online child sexual offences or are at risk of doing so to seek support (Walsh et al., 2023). Previous deterrence campaigns were informed by interviews with men who have accessed indecent images of children (Bailey et al., 2022). Findings from this previous study were translated into warning messages to deter the use of indecent images of children and encourage users to seek help (Walsh et al., 2023). The current study is an adaptation of the previous research, focussing on online grooming behaviour.

The objectives of the current study are to examine (1) reasons for offending reported by individuals who have engaged in online grooming behaviour, (2) the approaches they have used to establish contact with minors, (3) the pathways of their offending behaviour, (4) their appraisal of their offending over time, and (5) their opinions on what could help prevent offenders from (repeatedly) engaging in online sexual communications with minors. The findings reported below are based on the self-report of participants and their experiences of their online behaviours.

## Materials and Method

### Sample

Fourteen participants took part in this study. All interviewees were male. They had a mean age of *M* = 45.9 years (*SD* = 9.3, range = 30 – 62), and had 0-3 previous convictions (all previous convictions were non-sexual convictions). For further characteristics of the sample at the time of the interview, please see Table 1.

**Table 1:** Sample Characteristics

Relationship status	Any children	Employment status	Stage in criminal justice system
71.4% in a relationship 28.6% not in a relationship	42.9% have any children (including foster / adoptive children) 42.9% no children 14.3% unknown	57.1% employed / self-employed 35.7% unemployed 7.1% retired	57.1% under investigation 35.7% convicted 7.1% charged

### Procedure

Participants were recruited through the Lucy Faithfull Foundation (LFF), a UK charity dedicated to preventing child sexual abuse that runs the anonymous and confidential Stop It Now helpline for anyone with concerns about child sexual abuse. Callers to the Stop It Now helpline were made aware of this study if they have been under investigation for online sexual communications with/ about a minor; and clients of the LFF who received support through practitioners as part of assessments or interventions for online grooming were told about the research. Participants were made aware that they would not be anonymous to LFF if they take part in the research.

Participants received the information sheet and consent form and were encouraged to contact the lead researcher with any questions prior to signing the consent form. All participants gave informed consent, either by signing the consent form or giving verbal consent before the interview. Interviews were scheduled via email or over the phone. Interviews were conducted by full-time members of staff at the LFF, all of whom have a Master’s or doctoral degree and have experience in working with people who have sexually communicated with minors online.

Interviews lasted approximately one hour (mean length *M* = 61 minutes, *SD* = 19.8, range = 30-97 minutes) and were conducted over Zoom. Interviews were semi-structured and covered participants’ pathways to offending, their offending behaviour, and what they feel can be done to prevent online grooming (see Appendix 1, Supplementary Materials, for the interview schedule). Interviews were recorded and transcribed by the member of staff who conducted the interview. All participants received a debrief after the interview. Participants had the opportunity to withdraw their consent within seven days of the interview. No participant withdrew their consent.

This study received ethical approval by the LFF research committee.

### Analysis

Interviews were analysed using a ‘codebook’ thematic analysis approach (Braun & Clarke, 2006, 2021). The analysis was conducted by the first two authors. The first two authors read the transcripts several times to familiarise themselves with the data. They then generated initial codes and developed a codebook. Coding was done both deductively (analyst-driven) when factors previously described in the literature were identified, and inductively (data-driven) for additional factors not previously mentioned in the literature. Codes were primarily developed at a semantic level with limited interpretation of what participants had said.

Inter-rater reliability (IRR) was calculated for two of the transcripts (14% of all transcripts). IRR was calculated by dividing the numbers of agreements per transcript by the number of total amounts of codes used per rater in each transcript (see McAlister et al., 2017). IRR ranged from 57-79%. Disagreements involved the number of codes being used (for example, one author used longer sections of transcripts for one code whereas the other author split these sections into smaller segments, thereby generating lower number of codes being used; some codes had initially overlapping definitions and were therefore used interchangeably). All disagreements were discussed by the two raters, resulting in adjustments of the codebook and 100% agreement on the coding of the two transcripts. Both raters then proceeded to code the remaining transcripts individually using the codebook (see Appendix 2, Supplementary Materials).

After all transcripts had been coded, the two members of the research team identified initial themes, including candidate subthemes through discussion. Themes and subthemes were then checked and reviewed until homogeneity within themes and heterogeneity between themes was established. The final themes were then defined and named. Finally, analysis and findings were written up.

## Results

### Overview of Results

A short outline of the main findings is provided in this section. The themes and their subthemes are presented in more detail in the following sections. This short outline aims to provide readers with a summary of the main findings and to guide their reading of the following sections. Five themes were identified in the data and the two dimensions of *Risk factors*, i.e. factors relating to initiation, maintenance or facilitation of online grooming, and *Protective factors*, i.e. factors relating to the deterrence, disruption and prevention of online grooming, were present across all themes. The subthemes were grouped along these two dimensions (see Table 2).

**Table 2:** Themes and Subthemes

Themes	Social Aspects	Mental health / Psychological aspects	Justification for offending	Secrecy	Technology
Risk factors	Relationship and intimacy problems Risky social interactions	Negative experiences Negative Self-appraisal Stressors Unhealthy Coping Out of control behaviour Motivations	Disregarding Consequences Appraisal of behaviour Disconnection from Reality	Own secrecy Chat partner’s secrecy / identity	Legal behaviour Illegal behaviour
Protective factors	Positive relationships Social deterrents	Taking actions for recovery External support	Reality as deterrent Barriers for offending	Reducing anonymity / secrecy	Avoiding risky online behaviour

The first theme, *Social Aspects*, describes how participants’ problems in establishing or maintaining meaningful or intimate relationships offline led them to seeking out connections online and how certain risky interactions (e.g., with other adult chatters) facilitated sexual communications with minors. To promote desistance and prevent future offending, participants stressed the importance of trusting relationships offline and being around loved ones as a deterrent for risky behaviours.

*Mental Health / Psychological Aspects* subsumes negative life experiences, other stressors and unhealthy coping mechanisms that participants linked to their offending. This included a negative view of themselves and having engaged in escalating online behaviours. Proactive steps towards their own recovery, often coupled with support received from professionals, were identified as ways to build healthier coping strategies.

Participants discussed different *Justifications for Offending* that facilitated their risky or illegal online behaviours, including not considering the consequences of their behaviour and perceiving the online conversations as not real. Challenging these justifications, e.g. by reminding chatters of the reality of their behaviour and the people they chat with, may provide avenues for prevention of sexual conversations with minors.

The grooming behaviours were usually done in *Secrecy* and reducing one’s own anonymity, such as increasing the monitoring of chat platforms, may be beneficial in the prevention of online grooming behaviours.

Lastly, participants described their use of *Technology* that facilitated the offending behaviours, such as the wide accessibility of the internet, and that managing their online behaviours (e.g., stopping the use of social media) has helped them in desisting.

### Social Aspects

The *Social Aspects* theme involves how relationship problems and certain interactions with others can lead up to, trigger or facilitate online grooming, as well as how relationships and considerations of others may be helpful in preventing or deterring online grooming behaviour.

#### Risk Factors

Two subthemes were identified that relate to social risk factors for online grooming behaviour: *Relationship and Intimacy Problems* and *Risky Social Interactions*.

##### Relationship and Intimacy Problems

Participants described their difficulties with relationships and intimacy prior to or at the time of offending. This included being shy and insecure to talk to others or build relationships offline and therefore resorting to online communications. Feeling lonely and alone led participants to seek connection and interaction with people online. Online communications were experienced as easier and allowed participants to communicate more openly and comfortably (“I have always been isolated and suddenly I found this online world where I could be and say what I want”, Participant 11 [P11]). Loneliness was attempted to be combatted by interacting with people online to find some social interactions (“It was like a yearning for intimacy and, and just human interaction”, P1).

Some participants reported difficulties in being open and intimate with their partners, family and friends. Online communications enabled them to talk about issues they wished to discuss but found difficult to address with their loved ones face-to-face. Some participants approached minors because they felt that they were more open. Participants felt that this made an emotional connection easier with minors compared to adults: “I found that people who are younger give me that more in the sense that they’re less guarded. And then, and then in response I can be less guarded” (P6). Online communications therefore were used to meet relationship and intimacy needs that participants struggled to fulfil offline.

##### Risky Social Interactions

The online communications involved different forms of interactions, ranging from chit chat with other (perceived) adults to discussion of sexual topics with minors. Various risky social interactions were reported that related to sexual communications with minors.

Participants engaged in different approaches to establish contact with minors. A common method of starting conversations was contacting lots of chat users with short messages such as “Hi, how are you?” (P12) and then communicating with whoever responded, often including both adults and minors. Some participants made their intentions for sexual chat clear from the beginning, while others started with small talk. Participants often engaged in non-sexual communications with minors because they enjoyed the conversation and connection with others (see subtheme *Relationships and intimacy problems*) or to build trust before turning the chat into a sexual conversation.

I think for me it felt like we developed a friendship and we had general conversations. I think also it was an element of me trying to gain her trust. (P11)

To gain the trust of minors, some participants presented and communicated in a specific way with them. This included telling the minors that they themselves were teenagers, that they were photographers or model agents, or they took on a fatherly role in the communications with minors (“I lost my dad when I was quite young, myself, so maybe I tried to help these people, thought I could be somebody and it just escalated out of control”, P14).

In addition to these approaches to establishing online communication with minors, other communications with (perceived) adults were risky. One participant reported that other adult chatters encouraged him to use specific chat sites which led to the use of different chat services and eventually communications with minors.

#### Protective Factors

Having and building more *Positive (offline) relationships* and *Social deterrents* to online sexual communications were identified as Social protective factors.

##### Positive Relationships

Trusting relationships in which participants feel able to talk about their issues were repeatedly stressed as important support systems. Participants reported that having a trusted person to openly talk about their problems would have helped them stop offending. Although the arrest and detection of their offending had serious effects on participants and their loved ones (see *Social deterrents* subtheme), for some participants the arrest offered an opportunity to build stronger and healthier relationships. The detection of their offending forced them to be more open with loved ones and work through previous relationship issues (see *Relationship and intimacy problems* subtheme) to create more fulfilling relationships (“I think I’ve regained my relationship with my wife”, P3).

Having support from positive relationships has helped participants to desist from reoffending: “It was the fact I told probably 10-12 people you know, the unfiltered version and everyone was just (…) supportive, yeah. That made a huge difference” (P5).

##### Social Deterrents

At the time of offending, having people around was a deterrent for participants’ online behaviour. Participants reported that they tended to have online sexual communications when on their own, when their family or housemates were away or in a different room. Participants described the consequences of the arrest on their family and partners as one of the gravest impacts:

I mean I’ve lost my relationship with my wife (…) The hardest thing is probably the relationship with my daughter. Because, at the moment I can't be alone with her. That is incredibly difficult as a father to be in a position where people don't feel I am trusted. (P8)

Participants wished they had considered the consequences for their family before they offended to avoid causing them pain. However, considering the impact for their loved ones now helps participants desist from reoffending: “Think about the impact it will have on everybody involved with it (…) you know, it’s your wife, family, you know, everybody” (P3).

### Mental Health/ Psychological Aspects

The *Mental Health/ Psychological Aspects* theme centres around participants’ thoughts, feelings and behaviours that played a role in the commencement and maintenance of their offending behaviour and that have helped them move on from their offending to lead a more positive and healthy life.

#### Risk Factors

Six subthemes were identified relating to psychological risk factors for participants’ offending behaviour: *Negative Experiences* in their early life, participants’ *Negative Self-Appraisal*, *Stressors* at the time of offending, *Unhealthy Coping* styles, online behaviour that got *Out of Control*, and participants’ *Motivations* for online sexual communications with minors.

##### Negative Experiences

Participants described a variety of negative experiences that may have influenced their online behaviour. Negative life events in their childhood or adolescence affected participants’ mental health and participants struggled to cope and process these events. These events included early exposure to pornography, bereavement and difficult teenage years (“I never enjoyed any of my life really, never enjoyed my teenage years at all really hated them. So always feel like I wish I could go back to my teenage years”, P1).

Own sexual abuse and exploitation experiences also shaped participants’ appraisal of their behaviour. Participants said they learned to keep sexual experiences a secret due to their own abuse experiences or that they felt the inappropriate sexual behaviour was acceptable as this is what they had learned during their own abuse experience. One participant reported that he had been groomed online when he was a teenager. He described his own experience as “making me feel good as a kid” because the adult was “complimentary and very nice” (P5). Such experiences may shape people’s appraisal of their own behaviour.

##### Negative Self-Appraisal

A negative view and appraisal of themselves and their behaviour were commonly mentioned by participants. Some participants struggled with a lack of self-confidence which was related to negative experiences and/or their relationship and intimacy problems (see above). Sexual chats offered participants affirmation that helped them cope with their low self-esteem.

I think it all stems back to that first time when I was talking to that woman, (…) those feelings like validation and oh my God this person finds me attractive and it was insane. (P1)

Although participants have dealt with feelings of shame around their offending behaviour, they could distinguish this behaviour from their usual self: “It wasn’t me, but it was me but it wasn’t me. (…) It’s not the 99% me, it’s the 1% me” (P12).

##### Stressors

Before or at the time of their offending, participants were dealing with different stressors. Many participants mentioned stress, for example at work, as one of the main contributors to their offending behaviour. Emotional distress caused by relationship breakdowns and worries related to their loved ones was experienced by several participants. Participants reported that they struggled dealing with these issues at the time of their offending:

I mean I lost my [parent] (...) [Other parent] died with dementia so I had to cope with that. Then, my partner had a breakdown herself, my [sibling] had a cancer scare and a whole lot of things I've just put at the back of my head and try to deal with them all and I know now that I was a ticking time bomb. (P14)

Participants further tried to cope with their own mental and physical health problems. Sleep problems led to some participants distracting themselves with unhealthy internet use and offending behaviour.

##### Unhealthy Coping

Due to the negative experiences, negative self-appraisal and/or stressors, participants employed unhealthy coping mechanisms to deal with these worries and issues. Online pornography was used as a way of managing difficult emotions and mental ill health. Similarly, online sexual communications served as a way of coping with their problems by providing “a quick fix” (P14).

It always restarted at a point where there was something going on in my life. So be it to do with work. Or to do with my relationship, or lack of (…) Like it’s always filling a hole, always addressing emotional distress in an unhealthy way. (P6)

Some participants were struggling with their problems to an extent that they sought ways to escape their offline life. Therefore, they resorted to unhealthy online behaviour and offending behaviours: “I felt like I could go online and be this sort of completely other person or talk to people for hours about, just completely make up who I am.” (P1).

##### Out of Control Behaviour

The dysfunctional sexual coping (see above) culminated in a lack of control of participants’ online behaviour. Participants reported that they engaged in excessive internet use and an escalation of their pornography use online. Participants described how they became desensitised to legal adult pornography and then used increasingly more deviant and illegal material. For some participants, this led to an escalation to different harmful behaviours, including online grooming.

It's almost like I radicalized myself, I felt like I was getting more interested in younger looking models. (P6)

Many participants felt that they had been addicted to online pornography. Their pornography use was described as a habit or compulsion and participants experienced withdrawal symptoms when they stopped using pornography.

All driven by this “I need to, I need to look at some porn now”, “I’m feeling really, I feel really on edge, I need to have a look now”. (P13)

Participants described how their behaviour was so out of control that they felt no intervention was possible at the time of their offending and that the arrest was needed for them to stop (“I don't think I would have stopped. Yeah I think I needed that arrest.”, P2).

##### Motivations

In addition to their online behaviour being out of control, participants mentioned various motivations for their online grooming behaviour. This included dealing with boredom, enjoying the thrill of engaging in ‘taboo’ behaviour, and (sexual) excitement. Some participants described how they enjoyed feeling in control of the situation (“The excitement for me was the ability, I guess it's a control thing is probably the best way to summarize it, it's the ability to control the situation and control other people”, P9).

#### Protective Factors

*Taking actions for recovery* and *External support* were identified as subthemes covering protective psychological aspects to interrupt or prevent online grooming behaviour.

##### Taking Actions for Recovery

The Good Lives Model (Ward, 2002) was mentioned repeatedly as a framework that participants have used to help them maintain their desistance from offending and build a more positive future: “*My therapy has helped me understand the, well I guess framing it in the Good Lives Model, like understanding what a good life is, what was lacking and knowing (…) how to address that*” (P6). This has helped participants identify their needs, what was lacking at the time of offending, and how to fulfil these needs in a more prosocial way. Participants have taken steps to build a better life, including spending more time with their families, reducing stress at work and engaging in offline hobbies.

I’ve got tools of recovery in ways to help me, in things I do to get over those urges and get away from those urges and stop me from acting out. And every time I do those sorts of things, it does feel good. (P1)

I am trying to fill my time more productively. (…) I have started photography which I have been interested in years. I have started to read more books, some related to this and some general fiction. (P11)

Taking responsibility for their behaviour and the harm caused has also helped participants change their behaviour (“I think I’ve hurt too many people and it’s changed me and (…) everything about me”, P4).

Participants stated that the changes they have made to their lives have contributed to a reduced interest in dysfunctional sexual coping strategies as well as reducing the urge to engage in harmful online behaviour. These steps to recovery have given participants hope for the future. They estimate their risk for reoffending as low and feel they have turned their lives into a more positive direction: “It made me realize you just want to contribute back into society, but not to do this again so that's really a big factor in what's turned me around and I, you know I just don't want to do it anymore.” (P9)

##### External Support

External support has helped participants in taking steps to recovery. Only a few participants said they had spoken to someone about their offending before they were arrested. Most participants started searching for and accessing external support only after their arrest. Examples of support received after their arrest included Sex Addicts Anonymous, social workers and counselling/ therapy:

I saw a fantastic counsellor once a week and that was really helpful to have that kind of space where I could talk to someone about everything that was going on, and there was a lot going on in my life over those months. (P8)

All participants have engaged with LFF to address their offending behaviour. The online “Get Help”^1^1https://www.stopitnow.org.uk/concerned-about-your-own-thoughts-or-behaviour/ self-help modules on the Stop It Now website and the LFF psychoeducational courses for people arrested for online child sexual offences aided participants in reflecting on their behaviour, understanding the harms involved, and developing strategies to reduce their risk of reoffending.

In the early day, Stop It Now, the self-help modules really helped me reflect because anytime I reflected prior to this happening it would just lead to suicidal thoughts or feelings of negativity and just lead me to act out, so just negative circles. (P1)

Participants urged others in a similar situation to get external help as soon as possible (“Get help before you do anything. Seek help”, P4), and advised that support services should be advertised more to raise awareness in the general public and particularly amongst those at risk of offending. Such advertising should stress the non-judgmental attitude of the services to reduce the risk of stigmatisation and reduce barriers for affected people to contact the services.

Where such advertisements should best be placed was debated by participants. While some thought they should be online, for example on chat sites (see *Justification of Offending theme*), others thought a broader advertisement strategy would be more effective in reaching affected people. This could include using leaflets in surgeries of health services, adverts on TV or on busses. Participants thought that adverts should also be placed in a way that would not raise suspicions if people looked at them or picked them up.

### Justification for Offending

The *Justification for Offending* theme explores components that contribute to the participants’ internal justification for engaging in online grooming behaviour and factors that challenge this justification and contribute to deterring the offending behaviour.

#### Risk Factors

Three subthemes were identified that relate to increased justifications of the offending behaviour: little consideration for the C*onsequences* of their behaviour, justifying *Appraisal of* their own behaviour and the perception of *Reality* of the online conversations.

##### Disregarding Consequences

Most participants stated that they had little consideration of potential negative consequences while engaging in illegal online behaviour.

Participants acknowledged the illegality of the behaviour they were engaging in, but that this was ignored during the time of offending. One participant explained he was aware of the risk but did not expect police involvement: “You never think [the arrest is] gonna happen to you until obviously you get that [knock], so then reality kicks in”. (P9)

One participant described an initial concern about the negative consequences. However, this was easier to ignore as time went on without his illegal online behaviour being challenged: “As I said, this took place over a long period, so the more times that it happened, and I didn't get caught or nothing bad happened I became more relaxed.” (P8)

##### Appraisal of Behaviour

Participants described justifying their actions at the time of offending by internally appraising their online behaviour. The lack of awareness of the harm that was caused by their online behaviour was one factor that was described by participants.

There was no real physical person (…), so you felt there was no harm (…) you’re just talking to somebody, and it was, yeah it was very wrong but it’s not harming anybody. (P9)

During the time of offending, several participants reported that they did not perceive the chat as wrong. One participant was a victim of online grooming and had engaged in sexual chats from the age of 11. He explained that at the time of offending, he struggled to acknowledge the harm caused to the minor: “The fact I'd been in that shoe myself as a teenager, so in my mind, maybe I was phasing out the fact that it was wrong because I've done it myself.” (P5)

Other reasons why participants did not appraise their behaviour as wrong included considering the chats with minors as a friendship and not exclusively as a means for sexual gratification. This interfered with the ability to acknowledge the harm caused.

##### Disconnection From Reality

Participants described feeling a separation between the online sexual conversation and real-life interactions, making it hard to acknowledge the minor as a real individual. This contributed to the lack of awareness of the harm caused during the time of offending.

One participant stated that he would not end the conversation with the minor when confronted about their age as he did not acknowledge the conversation as real: “And so you know, in theory, again like I said I never thought people were real and still whenever they said they were said age, you know it was never a red flag, it would never stop the conversation.” (P12).

Fantasy or role-play were named as a factor that created a discrepancy between real life and the online chat. The chat was viewed as a method to fulfil their fantasy. “You know, initially you’re just typing letters on the screen, it’s not a real person, it made it very sort of disjointed from reality so became like a very much a fantasy.” (P9)

The separation between real life and fantasy becomes more apparent by looking at the participants’ intention to meet up with their minor chat partners. Thirteen out of 14 participants stated that they had no intention of meeting a minor face-to-face. This indicates that the disconnection from reality may be a risk factor for online grooming but a protective factor for contact sexual abuse.

#### Protective Factors

*Reality as deterrent* and *barriers for offending* were identified as subthemes covering protective factors to prevent offending and its justification.

##### Reality as Deterrent

Participants stated that acknowledging and understanding that their chat partner was a real person and that they are causing harm to an actual minor was a helpful tool to prevent reoffending (“Thinking about the people I was chatting to as real people, and what they're going through, what they might want to do (…) that's been the biggest thing for me”, P2).

The chat partner alerting to the illegality of their online behaviour was described as a constructive approach to stop chat from continuing (“It might have been as simple as having a conversation and someone then saying ‘Well actually, you know I’m going to go tell someone about this’.”, P8).

Another participant described an event where the chat partner mentioned circumstances in the real world, including their location and recent world affairs, which made him realise the reality of what the chat partner was describing in their chats and acknowledging that the chat was not purely fantasy. This acted as a deterrent for the participant and led him to block and report the chat partner.

##### Barriers for Offending

The participants identified several barriers to offending and to challenge justifications for offending. This included barriers that could prevent offending behaviour before it happens, interventions during the time of offending and factors that work as a deterrent to reoffending. Educating individuals earlier in life such as in school about the risk of pornography use and excessive use of chat rooms was identified as a beneficial approach to increase awareness and prevent offending. Increasing awareness of non-judgemental help available before police involvement was identified by participants as a barrier to persisting with the offending behaviour: “Knowing that there are people out there who are willing to help. You do feel very alone. It’s not like you are into football or Formula 1 and you can discuss it with people.” (P11)

Intervention during the time of offending, such as warning messages of chat sites, was proposed as a useful tool to deter online grooming behaviour. This may include alerting to the presence of minors on the chat sites, the harm sexual communications with minors are causing and the consequences of this behaviour.

I think had there been some kind of an intervention that wasn't the police arresting me. I honestly feel like that probably might have been enough. (…) Had someone gone “actually. I think you've got a problem here, and you need to deal with it and if you don't these could be the consequences.” (P8)

Thirteen out of 14 participants got in contact with Stop It Now after they had been visited by the police, and one participant had contacted Stop It Now prior to being detected by law enforcement. Participants reported that Stop It Now / LFF helped them understand their behaviour and that LFF contributed to recognising facilitating factor of their behaviour, justifications for offending, as well as tools, skills and support to avoid reoffending.

I think that made me think very deeply about a lot of things, but it also provided tools and ways to think about preventing those behaviours or preventing those kinds of unwanted thoughts. That was hugely helpful. (P8)

Taking responsibility for their action was viewed as a helpful tool for participants. This involved getting a further understanding of the harm caused to victims and taking responsibility for this consequence. Participant 8 stated that: “I think all of those consequences in terms of the victim I feel a huge amount of sadness about any impact I have had on real people and real girls.”

### Secrecy

The *Secrecy* theme explores how keeping secrets from others plays a part in offending behaviour and how reducing secrecy may help in preventing such harmful online behaviours.

#### Risk Factors

Risk factors in the *Secrecy* theme involved both O*wn secrecy* and C*hat partner’s secrecy / identity*.

##### Own Secrecy

Participants described living a secret online life and hiding offending behaviour from other family members and partners. The online grooming was kept secret and separate from participants’ real life. Participants, therefore, identified secrecy as a significant risk and maintaining factor for their offending behaviour.

Keeping secrets (…) being excited by the taboos, whatever that may be. (...) there's many taboos that generally you keep that secret to yourself. (…) if I knew of somebody who's very secretive, I think I think they could possibly have problems in the future. (P3)

The stigma associated with online grooming was explained by participants as an obstacle to seeking help from professionals or from individuals they have a close relationship to. The stigma deterred participants from opening up about their secretive behaviour.

Secrecy was also prevalent in the online chat. The level of discrepancy between real identity and online identity varied between participants. However, most participants withheld or altered some of the information they provided to their chat partners. This included not providing their actual name or age and using a different profile picture. This enabled participants to be anonymous online, disinhibiting them to illegal behaviour.

##### Chat Partner’s Secrecy / Identity

The chat partner’s secrecy / identity refers to the participants’ perception of their minor chat partner and whether they were being truthful during the chat. The level of suspicion on the accuracy of the details provided by the minor varied between participants. Some participants did not acknowledge or “register” (P9) the age of their chat partners. Others expressed that they did not expect any chatters on the sites to give any accurate information. This caused them to disregard information provided by the chat partner and to engage in sexual communications.

I never thought people were real and still whenever they said they were said age, you know it was never a red flag, it would never stop the conversation. (P12)

Most participants were initially detected by law enforcement due to a police decoy. This was used as a justification for the offending behaviour by some participants as the chat partner had provided false details and thus was not a “real” minor. Knowing or assuming that the chat partner is not a real child due to secrecy of the chat partner may therefore be a facilitating factor in online grooming behaviour. (“I got caught, because the person I was chatting to wasn't who they said they were, they were a police officer. So in in my head and kind of again, justifies it to me in a strange way that never people were who they say they were. They just weren’t.”, P12).

#### Protective Factors

One subtheme was identified for the protective factors theme: *Reducing anonymity/secrecy.*

##### Reducing Anonymity / Secrecy

Measures were proposed by participants for both individual and external factors that could assist to reduce the reality or appearance of secrecy and anonymity on online chat platforms. Increasing the monitoring and control of chat sites was identified as a factor to potentially deter online grooming behaviour, for example by monitoring the age of individuals using the platforms and reducing anonymity by having individuals provide some identifying information about themselves.

There's so little control (…). Unless you log it back to some of these Facebook account or something (…) I know people can create fake accounts, (...) tie it into something you know, to make it a little bit more (…) of a validity check. (P9)

Other suggestions included reducing the anonymity of the chat platforms through visible supervision of the site, for example by the police. Having a family member or partner control the participants’ internet use was recognised as a protective measure to hinder illegal online behaviour in the home setting by decreasing secrecy. Such a step would also improve the communication and honesty in their close relationships and act as a safety net to avoid offending behaviour.

### Technology

The last theme entails participants’ use of technology and the internet that was related to their offending behaviour.

#### Risk Factors

Risky technology use involved both *Legal behaviour* and *Illegal behaviour*, which constitute two subthemes of the Technology theme.

##### Legal Behaviour

Participants used the internet for a multitude of ways, such as for checking the news, emails, shopping or streaming of films and series. For some participants, this culminated in excessive internet use (see *Mental Health / Psychological Aspects* theme). After the arrest, participants reported a decrease in their overall internet use.

Self-reported technological knowledge varied in the sample. While some participants described themselves as “pretty tech savvy” (P6), others reported a lack of technological knowledge: “Oh god, compared to the kids nowadays: useless. Can I have an L plate?” (P7). Despite some technological knowledge, participants did not engage in extensive efforts to conceal their identity using technological means like the use of VPNs.

Accessibility of the internet, online pornography and chat sites presented a risk for participants, facilitating excessive and harmful online behaviour without creating barriers for their offending. Participants used various legal websites and apps to access pornography and online chats. This included websites hosting pornography videos and/or erotic stories. Messaging apps were used to access both adult pornography and chats.

##### Illegal Behaviour

Like their use of technology and the internet for legal purposes, participants used technology for their offending behaviour. Various websites and apps were used to share images, including indecent images of children.

I went onto [app] and after a little while of communicating with people and things like that. I found my way into an underage group and on that group, people basically shared videos and images of underaged people. (P11)

Some participants moved between platforms to facilitate different forms of illegal behaviours. Webcam chat platforms were used to move online chats with minors onto webcam and interact with the minor in real time while being able to see them.

If chat platforms did not facilitate the exchange of images, participants reported using other websites that allowed image sharing so that participants could exchange images with their minor chat partners. The dark web was mentioned by only one participant as a means of accessing images.

When participants were aware of the illegality of their behaviour (see *Justification for Offending* theme), they at times adapted their use of technology to avoid detection: “There were always things happening on the [app] groups where people would disappear suddenly. You always wondered what had happen and why that was. If [app] had discovered it and closed it down, which is why I used the pay as you go phone.” (P11)

#### Protective Factors

*Avoiding Risky Online Behaviour* was identified as a subtheme of the Technology theme covering participants’ behaviour and use of technology (or lack thereof) to support their desistance.

##### Avoiding Risky Online Behaviour

Participants who initially chatted online with (perceived) adults reported that they were aware of the illegality of sexually communicating with minors (see *Justification for Offending* theme). This deterred them initially from engaging in online communications with minor chatters and they took steps to avoid communicating with (perceived) minors: “So, if (…) somebody literally walked into the chat room and said, I am a 13-year-old girl, for example, or a 13-year-old boy, then, no, no, no, no.” (P7)

The arrest and legal proceedings stopped participants’ offending behaviour and helped them recognise their own risk factors. Participants reported that they have since adapted their technology use. They reduced their use of online pornography significantly since the arrest, stopped using social media, or limited their internet use (see *Out of Control Behaviour* subtheme) as much as possible: “I’ve gone old school mobile phone with buttons (…) but yeah that is essentially my internet safety plan.” (P2)

In some cases, participants’ technology and internet use is further restricted by court orders, for example, ordering participants not to use certain online platforms or delete their browsing history.

## Discussion

This study examined pathways into online grooming offending and approaches to prevent and disrupt such behaviour. Five themes were identified that were all related to the onset, facilitation and maintenance of online grooming behaviour as well as to its deterrence, disruption and prevention. Relationships and social aspects, mental ill health and psychological factors, engaging in justifications for online offending, being secretive about online identity and behaviour, and the use of the internet and certain technology are all related to the commission of online grooming behaviour and its prevention.

These factors correspond with findings of other studies on online child sexual offences. Based on Ward’s (2000) earlier work on contact sex offenders’ implicit theories, Paquette and Cortoni (2022) identified offence-supportive cognitions in people who had committed online child sexual offences, including online grooming offences. The authors found that online offending may be related to perceiving the offline and adult *world as dangerous* and therefore children as less threatening to interact with; in addition, children may be considered an equal *partner* in the conversation or relationship. Further, the authors identified the *uncontrollability* of external circumstances, such as stress or illness, and specifically the *uncontrollability of the internet* (e.g., highly accessible) as offence-supportive cognitions. Participants in the current study spoke about similar psychological risk factors, including stressors and addictive online behaviours. Paquette and Cortoni’s (2022) theme *Virtual is not real* was corroborated by the account of the current sample of not perceiving their online behaviour as real. In line with the current finding, Paquette and colleagues (2023) found that over half of their sample of people who have engaged in online grooming (*n* = 38) endorsed this cognition, indicating that this may be a common offence-supportive cognition among people who have engaged in online grooming.

**Figure 1:**
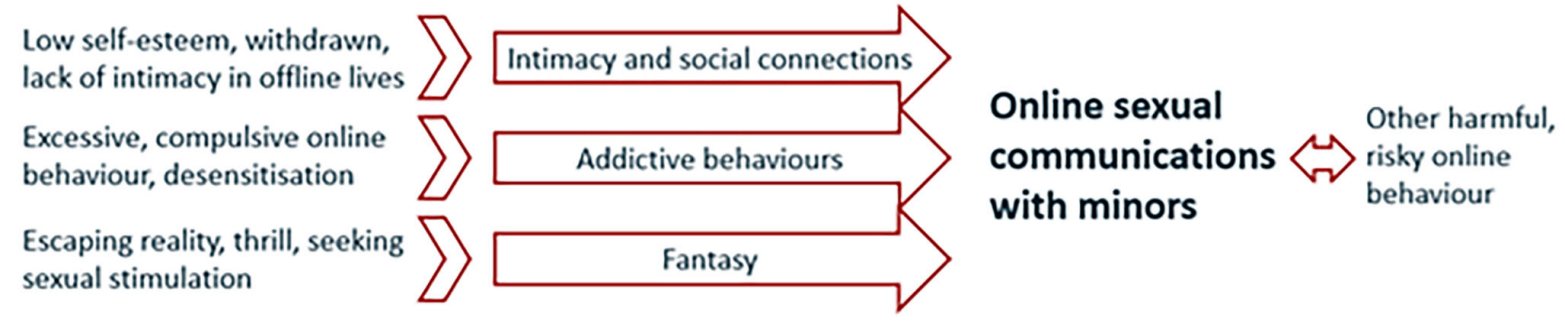
Three Hypothesised Pathways Into Online Grooming Behaviour

The current findings indicate that there appear to be different pathways into online grooming (see Figure 1). *First*, some participants had sexual communications to find a social connection because they struggled with relationships and intimacy in their offline lives (cp. Middleton et al., 2006). Online communications were perceived as safer and easier to communicate more openly and find an emotional connection. This pathway was identified in participants who reported a lack of confidence and appeared socially withdrawn. The *second* pathway relates to addictive online behaviour and online behaviour that is considered out of control (cp. Bailey et al., 2022). Excessive internet use and compulsive or habitual online pornography use may lead to a desensitisation to adult pornography. New sexual material and other online sexual behaviours may therefore be used to achieve (sexual) gratification or manage withdrawal symptoms. This may involve online child sexual exploitation behaviour including online sexual communications with minors. The *third* pathway involves adult chatters who engage in online behaviours to escape reality and engage in (sexual) fantasy role play, seeking sexually stimulating conversations with others. These behaviours are experienced as a disassociation from reality with the screen creating a boundary between the chatters making them feel not real (cp. Paquette & Cortoni, 2022). The anonymity of the internet facilitates this experience of chats as purely fantasy.

The online grooming behaviour may then be related to other harmful or risky online behaviours, including the use of child sexual abuse material. Risk factors for additional illegal online behaviours may be problematic internet use (Morgan & Lambie, 2019) and a sexual interest in children (Seto et al., 2006). However, the link between online sexual communications with minors and contact sexual offending seems less straightforward. Only one participant in the current sample said that he was planning to meet with the minor he was talking to online. The rest of the sample reported no interest in meeting with a child. This is in line with previous research showing that people who offend online have a low risk of engaging in offline sexual offences (Elliott et al., 2019). The current suggested pathways and the relationship between online grooming behaviour and other harmful or illegal online and offline behaviours should be further explored in future research.

Findings from the current study may inform new ways to deter, disrupt and prevent online grooming behaviour. Participants advocated for more warning messages and supervision or monitoring of online chat platforms to deter or disrupt online communications with minors. The anonymity of the internet and lack of supervision may lead to the internet being perceived as a *lawless space* (Steel, 2021) by those who engage in illegal online behaviours. Situational prevention efforts like warning messages may therefore be effective in reminding platform users of the harmfulness or illegality of certain behaviours and consequently deter them from engaging in these behaviours.

Warning messages on pornography websites are an avenue for preventing and disrupting the use of illegal or ‘barely legal’ material (Prichard, Wortley, et al., 2022). Similarly, artificial intelligence chatbots appear promising in helping to disrupt the use of child sexual exploitation material (Prichard, Scanlan, et al., 2022). Based on these research findings and the responses by participants in the current study, such approaches may also be worth exploring in more detail for online grooming behaviour.

Increased supervision or monitoring of chat platforms may involve the suspension of users. A recent study examining the effects of suspension on offensive and offending users of a Japanese chat app found that suspensions of users decreased the risk for further harmful behaviour on the app, not just by blocking the user in question but also by reducing the risk for harmful behaviour in the user’s peers online (Yokotani & Takano, 2022). Whether the suspension of adult chat platform users engaging in risky or harmful behaviours may have a similar positive effect on increasing the safety on such platforms should be investigated in future research.

The situational prevention efforts on online platforms proposed by participants, including increased monitoring, align with the recently passed UK Online Safety Act 2023. This new Act places responsibility on online platform providers to keep users safe from illegal and harmful content. The proposed prevention efforts raised by this study offer some suggestions of how the regulations and responsibilities of online platforms set out by Online Safety Act 2023 could be implemented.

In addition to these situational deterrence and disruption approaches, current findings indicate that additional support for people at risk of engaging in online grooming behaviour is needed. Participants called for more advertising of available support services, echoing previous findings with users of indecent images of children (Bailey et al., 2022). Opportunities to speak to someone who offers support and reacts in a non-judgemental way may help people stop ongoing problematic online behaviours. However, in some cases intervention by law enforcement may be needed to stop behaviour that the person feels they have lost control over.

Assisting individuals to move on from their offending behaviour and directing them onto more positive lifestyle that allows them to meet their needs in a healthy and prosocial way appears an effective way to encourage and maintain desistance. The Good Lives Model (Ward, 2002) was pointed out through the findings as a useful tool to desist from online grooming behaviour. The model focuses on meeting “primary goods” which are unique to the individual in terms of importance and focuses largely on interests, abilities and the individual's future ambitions and goals. The participants pointed out how the adherence to these principles resulted in a twofold effect. It not only diminished their inclination to maladaptive coping mechanisms but had also acted as a deterrent against engaging in online grooming behaviour. As a result, participants got a newfound sense of optimism for their prospects and a reduced belief that they will reoffend.

### Limitations

The study had several limitations which should be considered. Firstly, all participants were LFF clients who had self-referred to Stop It Now for advice and support to help them understand and change their behaviour. All participants had agreed to be contacted for research purposes, therefore being a self-selecting sample. All participants expressed that they knew their behaviour was inappropriate. Therefore, the findings may not be applicable to individuals who did not believe they were acting inappropriately or illegally at the time of offending.

Participants were interviewed by LFF staff. They might have already established positive relationships with LFF staff and responded in a socially desirable way, particularly when speaking positively about LFF and Stop It Now. However, participants were informed that their participation would have no impact on their involvement with and treatment by LFF.

The self-report given in the present interviews refers to detected online grooming offences. The findings may hence not extend to undetected offending behaviour. Undetected offending may, for example, take place on different online platforms and undetected offenders may engage in different approaches to reduce risks for detection.

### Implications and Future Directions

The findings of this study have important implications for the prevention and deterrence of first-time and repeated online child grooming offences. Wider education and access to support services for people at risk of offending may prevent first-time offending. Support services should therefore be more widely advertised. Situational prevention and disruption efforts may help prevent first-time and repeated offending. Chat platforms may use warning messages or splash pages to deter sexual communications with minors. Reducing chatters’ anonymity, for example by asking for verification of their age, may lower the risk for minors to use adult chat rooms and adult chatters to use teen chat rooms. New technological approaches may be tried to deter people from online grooming behaviour for example chat bots that alert adult chatters to the harmfulness of sexual communications with minors.

Any approaches employed to prevent and deter online grooming behaviour should be evaluated for their effectiveness. Due to the ever-changing online landscape, trends in online child grooming behaviours need to be constantly investigated to adapt existing prevention and deterrence efforts and develop new ones to respond effectively to new developments.

## Supplementary Materials

The Supplementary Materials contain the online appendices for this article (for access, see Wefers et al., 2024):

- Appendix 1 is the interview schedule used for this study.
- Appendix 2 is the codebook used for the analysis of this study.

WefersS.
DiesethT.
GeorgeE.
ØverlandI.
JolaparaJ.
McAreeC.
FindlaterD.
 (2024). Supplementary materials to "Understanding and deterring online child grooming: A qualitative study"
[Online appendices]. PsychOpen. 10.23668/psycharchives.14139
PMC1177873939886042

## Data Availability

The data that support the findings of this study are available from the corresponding author, SW, upon reasonable request.
